# Asymmetric Hybrid Polymer–Lipid Giant Vesicles as Cell Membrane Mimics

**DOI:** 10.1002/advs.201700453

**Published:** 2017-12-05

**Authors:** Ariane Peyret, Emmanuel Ibarboure, Jean‐François Le Meins, Sebastien Lecommandoux

**Affiliations:** ^1^ Laboratoire de Chimie des Polymères Organiques LCPO Université de Bordeaux CNRS Bordeaux INP UMR 5629 16 Avenue Pey Berland F‐33600 Pessac France

**Keywords:** asymmetric vesicles, biomimicry, cell membrane, copolymer, lipid

## Abstract

Lipid membrane asymmetry plays an important role in cell function and activity, being for instance a relevant signal of its integrity. The development of artificial asymmetric membranes thus represents a key challenge. In this context, an emulsion‐centrifugation method is developed to prepare giant vesicles with an asymmetric membrane composed of an inner monolayer of poly(butadiene)‐*b*‐poly(ethylene oxide) (PBut‐*b*‐PEO) and outer monolayer of 1‐palmitoyl‐2‐oleoyl‐*sn*‐glycero‐3‐phosphocholine (POPC). The formation of a complete membrane asymmetry is demonstrated and its stability with time is followed by measuring lipid transverse diffusion. From fluorescence spectroscopy measurements, the lipid half‐life is estimated to be 7.5 h. Using fluorescence recovery after photobleaching technique, the diffusion coefficient of 1,2‐dioleoyl‐*sn*‐glycero‐3‐phosphoethanolamine‐*N*‐(lissamine rhodamine B sulfonyl) (DOPE‐rhod, inserted into the POPC leaflet) is determined to be about *D* = 1.8 ± 0.50 μm^2^ s^−1^ at 25 °C and *D* = 2.3 ± 0.7 μm^2^ s^−1^ at 37 °C, between the characteristic values of pure POPC and pure polymer giant vesicles and in good agreement with the diffusion of lipids in a variety of biological membranes. These results demonstrate the ability to prepare a cell‐like model system that displays an asymmetric membrane with transverse and translational diffusion properties similar to that of biological cells.

## 1 Introduction

The mimicking and reproduction of natural components or systems is a fantastic source of inspiration and innovation in materials science from many decades.^1^, ^2^, ^3^ Among these biomimetic systems, cells are certainly not only the most fascinating but also the most difficult to reproduce both from structural and functional view points. Some key properties have been reproduced, such as compartmentalization,^3^, ^4^, ^5^, ^6^, ^7^, ^8^, ^9^, ^10^, ^11^, ^12^, ^13^, ^14^ cytoskeleton mimics,^15^, ^16^, ^17^, ^18^, ^19^ or simple (bio)chemical reactions.^20^, ^21^, ^22^, ^23^, ^24^, ^25^, ^26^, ^27^, ^28^, ^29^ The cell membrane structure is often simplified using lipids, or lipid mix with addition of cholesterol, which is really far from reality, not only considering the chemical structure but also the biophysical properties of the membrane.^30^ Another important feature that is often neglected concerns the asymmetric composition of the membrane.

As such, the concept of lipid bilayer asymmetry was introduced shortly after the idea of the fluid mosaic model to describe biological cell membranes.^30^, ^31^, ^32^, ^33^ The two leaflets of the membrane are structurally and functionally different in many aspects, this heterogeneity being crucial in maintaining cell activity and cellular events. The main source of asymmetry in cell membranes resides in the lateral and transversal heterogenous distribution of lipids between both sides. For instance, choline derivatives such as phosphatidylcholine or sphingomyelin are exposed in higher proportions on the external monolayer. On the other hand, negatively charged lipids such as phosphatidylserine (PS) are located mainly on the cytoplasmic side.^31^, ^32^ This uneven distribution of lipids affects membrane physical properties such as curvature, stability or permeability and warrants efficient signal transduction.^33^ In addition, asymmetry is a consequence of differing enzymatic activities between both sides of the membrane and also results from the positioning and orientation of membrane proteins, such as glycoproteins that are located on the outer leaflet and are involved in cell recognition.^34^, ^35^ The proper functioning of membrane‐regulated cellular phenomena is highly influenced by the asymmetric positioning of membrane constituents and therefore, a lot of energy is invested to maintain it. Perturbation or breakdown of asymmetry often has significant physiological consequences.^36^ For example, PS exposure on the outer leaflet is a sign for cell death and recognition by macrophages.^37^, ^38^, ^39^ While many aspects of cellular membrane asymmetry have been unveiled, how asymmetry is assembled and maintained as well as its full implication in membrane‐regulated cellular events is still not entirely understood. Hence, it is of major interest to better understand the importance of membrane heterogeneity and one way to do that is by developing asymmetric cell‐like biomimetic systems with well‐defined membrane properties.

In order to study and better understand the structure and function of biological membranes, model artificial bilayer asymmetric membranes have been prepared, in the context of cell biomimicry.^40^, ^41^ There are only a handful of reports on the preparation of such systems, mainly because of the difficulties to control and characterize the asymmetry. Different approaches have been considered, mostly through the fabrication of supported bilayers (i.e., bilayer on a solid surface) or the preparation of vesicles. A number of techniques have been developed to afford asymmetric lipid vesicles (so‐called liposomes) such as microfluidic devices^42^, ^43^, ^44^ or droplet‐transfer over an interface^45^, ^46^ while supported asymmetric lipid bilayers have been prepared mainly through vesicle fusion^47^, ^48^ or lipid exchange techniques.^49^ However, other types of amphiphiles namely peptides, polymers or lipid/polymer hybrids were also used to afford various structures, respectively nanoribbons,^50^ polymer vesicles^51^, ^52^ or tubular vesicles^53^ with an asymmetric membrane. While all these systems allow new insight into bilayer asymmetry and how it impacts membrane physical properties, there still is a need to further push the frontiers of cell biomimicry by developing stable systems with higher control of membrane properties. Many challenges still need to be addressed. For instance, supported bilayers are limited as models of cell membranes as compared with vesicular structures.

Combining the advantages of polymer chemical versatility and robustness with lipid biocompatibility is an interesting way to modulate and mimic cell membrane properties. The association of block copolymers and phospholipids is a relatively recent approach that have been developed to design bioinspired vesicular structures whose membrane properties could be modulated by composition and membrane structuration.^54^, ^55^, ^56^ These systems, so called giant hybrid polymer–lipid unilamellar vesicles (GHUV) or large hybrid polymer lipid Vesicles (LHUV) are especially investigated to generate lateral heterogenous distribution of the components (formation of “raft‐like” nanodomains of lipids),^57^ but to our knowledge the association of lipids and polymers has never been investigated so far to develop entirely asymmetric membranes.

We herein introduce a versatile method to produce asymmetric giant hybrid polymer–lipid unilamellar vesicles (aGHUV). In the present work, the vesicles are constituted of an inner leaflet of poly(butadiene)‐*b*‐poly(ethylene oxide) copolymer and outer leaflet of lipid type via an emulsion‐centrifugation method. We also show how we can prepare the reverse structures with the lipid leaflet facing the interior of the vesicle. We demonstrate the asymmetric character of the membrane and follow its stability by fluorescence quenching measurements. In addition, we further investigate lipid dynamic responses such as lateral and transverse diffusion (flip‐flop). We thus provide an efficient method to afford aGHUV that exhibit close resemblance to the architecture and membrane diffusion dynamics of biological cells. This system could serve as a tool and scaffold to better understand the importance of asymmetry and how it is maintained in biological systems.

## 2 Results and Discussion

**Figure 1:**
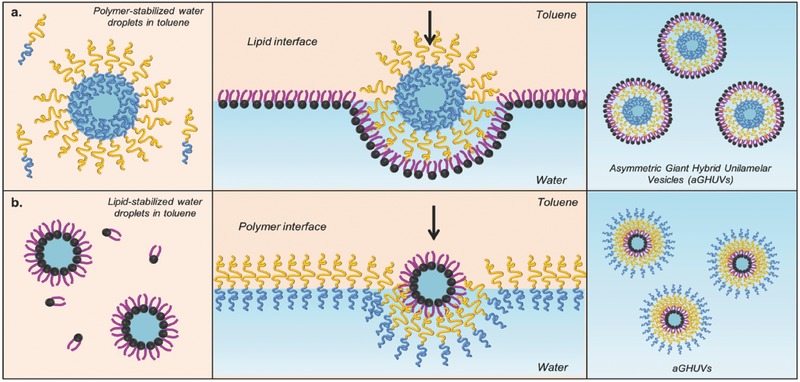
Schematic representation describing the preparation of asymmetric giant hybrid unilamellar vesicles (aGHUV). a) Preparation of aGHUV with an outer monolayer of lipid and inner monolayer of polymer and b) reverse aGHUV with an outer monolayer of polymer and inner monolayer of lipid. The scheme represents the aGHUV obtained just after formation.

A previously reported emulsion‐centrifugation protocol^58^ was adapted to afford asymmetric giant hybrid unilamellar vesicles with an aGHUV consisting of an inner leaflet of poly(butadiene)‐*b*‐poly(ethylene oxide) (PBut_2.5_‐*b*‐PEO_1.3_) and an outer leaflet of 1‐palmitoyl‐2‐oleoyl‐*sn*‐glycero‐3‐phosphocholine (POPC) lipid. Briefly, an emulsion of sucrose droplets in toluene stabilized by PBut_2.5_‐*b*‐PEO_1.3_ was poured over a POPC‐stabilized glucose/toluene interface at room temperature (25 °C). One should note that the temperature should be above the lipid transition temperature (−2 °C for POPC) for the lipid to efficiently stabilize the interface. With help of centrifugation and the difference of density between sucrose and glucose, the polymer‐stabilized sucrose droplets cross the interface while getting coated with an outer monolayer of lipid, and the resulting POPC/PBut_2.5_‐*b*‐PEO_1.3_ aGHUV can be recovered in the lower glucose phase (**Figure** **1**a). Additionally, as shown in (Figure 1b), the reverse aGHUV with an outer leaflet of polymer can be obtained. However, owing to the difficulties to form stable lipid‐stabilized emulsion droplets, the vesicles were formed in a rather low yield, which made the characterization of the membrane asymmetry more complex (see Figure S1, Supporting Information).

**Figure 2:**
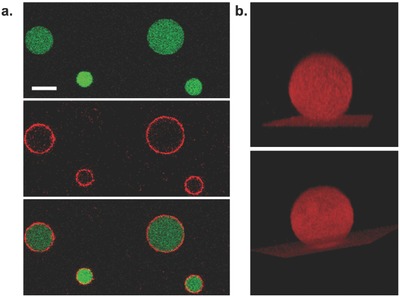
Confocal observations of POPC/PBut_2.5_‐*b*‐PEO_1.3_ asymmetric vesicles. a) The membrane is tagged with DOPE‐rhodamine (red) and the vesicles are loaded with fluorescein (green). Top: emission of fluorescein; middle: emission of rhodamine; bottom: overlay. Scale bar: 10 µm. b) 3D reconstruction of an asymmetric vesicle (≈20 µm diameter). Two different views of the same vesicle (red channel). Videos are available in the Supporting Information (Videos S1 and S2).

Examples of hybrid asymmetric vesicles are scarce and most reported systems consist of a bilayer formed from two different lipid types^42^, ^43^, ^44^, ^45^ or two different polymers.^51^, ^52^ To our knowledge there is only one reported example of such a system, but no clear evidence was provided to fully support the membrane asymmetry because of the impossibility to perform a fluorescence quenching assay.^45^ The lipid‐stabilized interface induced exposition of the lipid monolayer on the external side while the polymer monolayer is on the internal side of the vesicle. POPC was first chosen as a model lipid because it is one of the most represented lipids on the exoplasmic side of human red blood cells membrane and we hypothesized that it would add more flexibility and permeability as compared to a pure polymer membrane.^59^ Similarly, keeping a polymer leaflet should provide increased stability and influence membrane diffusion properties in contrast with a purely lipidic bilayer. For confocal observation, the vesicles were loaded with fluorescein and the membrane was tagged with 0.1 wt% DOPE‐rhodamine (DOPE‐rhod, λ_exc_ = 561 nm), which has a preferential positioning in the lipid phase (**Figure** **2**a,b). The images show a homogeneous red membrane and a vesicle population with sizes ranging from 10 to 30 µm. This homogeneous distribution of the lipid can be qualitatively interpreted as a first sign of membrane asymmetry (see Videos S1 and S2 in the Supporting Information for clarity).

**Figure 3:**
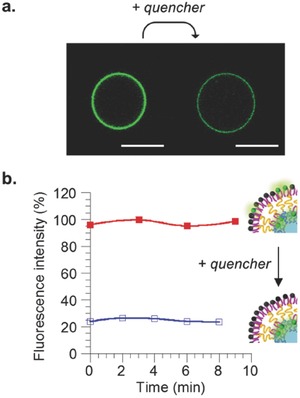
Spectral and microscopy analysis of POPC/PBut_2.5_‐*b*‐PEO_1.3_ asymmetric vesicle solution incorporating PE‐NBD on both sides of the membrane, before and after addition of dithionite as fluorescence quencher. a) Confocal microscopy observations before (left column) and after (right column) addition of dithionite. Scale bar = 10 µm. b) Fluorescence intensity of vesicle solution before (▪) and after (□) addition of quencher.

In order to demonstrate the asymmetric repartition of lipids in the membrane, a small fraction of 1,2‐diphytanoyl‐*sn*‐glycero‐3‐phosphoethanolamine‐*N*‐(7‐nitro‐2‐1,3‐benzoxadiazol‐4‐yl) (PE‐NBD, λ_exc_ = 488 nm) was added to the lipid interface in order to recover it in the external lipid monolayer after vesicle formation. NBD is a fluorescent dye that emits in the green and can be reduced upon reaction with sodium dithionite leading to a complete loss of fluorescence.^60^ A total loss of fluorescence from the vesicles after addition of dithionite to the solution would indicate 100% asymmetry assuming that the quencher does not cross the membrane. To verify this hypothesis, we added a small fraction of PE‐NBD in the PBut_2.5_‐*b*‐PEO_1.3_ solution in toluene used to form the emulsion as well as in the POPC solution for the interface. After vesicle formation, the green‐tagged lipid was consequently present in both leaflets and the aGHUV could be visualized under confocal microscopy (**Figure** **3**a).

Immediately after addition of the quencher (final concentration: 60 × 10^−3^ m), a decrease of fluorescence intensity was systematically observed for all vesicles. This decrease came from the reduction of NBD on the lipid outer leaflet of the membrane, which resulted in an extinction of the fluorescence. The remaining fluorescence observed could be attributed to the PE‐NBD inserted in the inner polymer monolayer. We further quantified the fraction of tagged lipid in each leaflet by means of fluorescence spectroscopy (Figure 3b). Fluorescence of the tagged‐vesicle solution was monitored over time before and after addition of dithionite. The results confirmed the confocal observations and showed that the major fraction of PE‐NBD was inserted into the lipid monolayer during vesicle formation while the rest remained in the polymer inner layer. The fact that we did not observe a complete loss of fluorescence confirmed that dithionite does not cross the membrane most likely because of its negatively charged character that is known to prevent membrane penetration.

**Figure 4:**
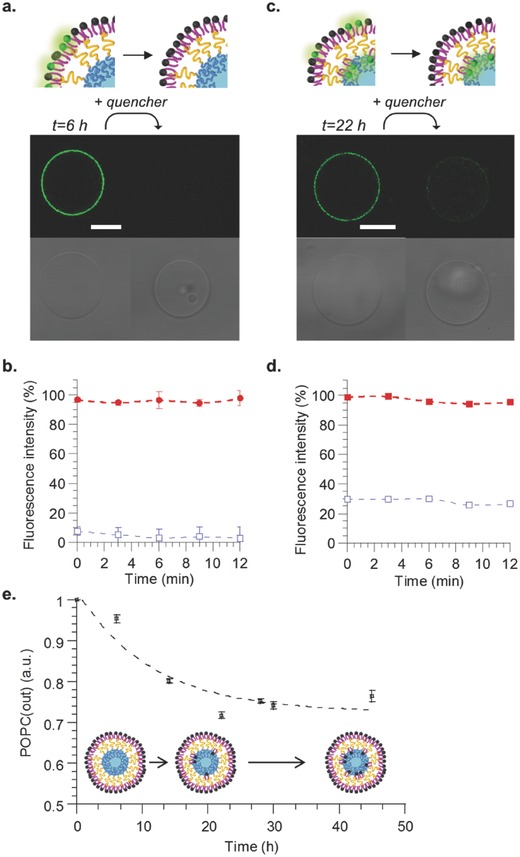
Spectral analyses and microscopy observation of asymmetric vesicles as function of time. Confocal microscopy observations a) after 6 h and c) after 22 h before (left column) and after (right column) addition of dithionite quencher. Top images: NBD emission, bottom images: white light. Scale bar = 10 µm. Fluorescence intensity of vesicle suspension b) after 6 h and d) after 22 h, before (▪) and after (□) addition of quencher. e) Kinetics of POPC transverse diffusion from the outer to the inner leaflet of the membrane. Measurements were performed in triplicate at 25 °C. For every time point, data were normalized with the total fluorescence intensity of the vesicle suspension before addition of the quencher.

To further demonstrate the bilayer asymmetry, aGHUV vesicles were prepared with addition of a fraction of PE‐NBD only in the lipid solution. One can hypothesize that if the vesicles are completely asymmetric, with an inner monolayer of polymer and outer monolayer of lipid, the fluorescence signal should completely disappear upon addition of the dithionite quencher to the vesicle solution. On the contrary, if lipids distribute symmetrically, or if a portion goes into the polymer phase, the fluorescence intensity should only decrease but not completely disappear upon reduction of the NBD dye. It has been shown indeed in the case of GHUV and LHUV that part of the lipid can go into the polymer phase (this can be simply observed using epifluorescence and confocal microscopy) and the partition coefficient have also been quantified using time resolve fluorescence spectroscopy techniques in LHUV.^57^, ^61^, ^62^ **Figure** **4**a shows confocal images taken 6 h after vesicle formation. Before addition of the quencher, the vesicles presented a homogeneous green membrane upon excitation at 488 nm. The addition of sodium dithionite to the vesicle solution (final concentration: 60 × 10^−3^ m) resulted in a complete disappearance of the fluorescence signal, attesting both the unilamellarity and the asymmetric character of the vesicle membrane. In order to get more quantitative measurements on an ensemble of vesicles, the same experiments were performed by fluorescence spectroscopy: no residual fluorescence after addition of the quencher could be detected, confirming the complete asymmetric character of the membrane, with the lipid constituting the external leaflet of the membrane (Figure 4b). Indeed, if vesicles were not asymmetric or if they would be multilamellar, a residual fluorescence should be observed, which is not the case. To demonstrate the versatility of our protocol, the same experiment was conducted with 1,2‐dimyristoyl‐*sn*‐glycero‐3‐phosphocholine (DMPC) instead of POPC, (see Figure S2, Supporting Information). Again, a complete loss of fluorescence upon addition of sodium dithionite was observed, confirming the membrane asymmetry of the formed vesicles. It seems that although lipid partitions partly in polymer rich phase in GHUV obtained by electroformation (via lateral diffusion), for this protocol, a partial migration of the lipid in polymer phase is limited, probably because it implies transverse diffusion at least few hours after formation of the vesicles.

This transverse diffusion, also called flip‐flop, is an important characteristic of biological membranes and resides in the ability of lipids to move from the exoplasmic side to the cytoplasmic side and vice versa.^63^ This is energetically unfavorable and happens at rather slow rates and with the help of enzymes. Flip‐flop rates have been evaluated for different types of lipids and lipid bilayers in several synthetic systems such as supported bilayers or large unilamellar vesicles.^44^, ^47^, ^64^, ^65^, ^66^, ^67^ However, owing to the much larger sizes of biological cells, membrane properties such as curvature (and thus lipid diffusion rates) would be more accurately represented by giant vesicle systems. We thus investigated POPC trans‐bilayer diffusion on the aGHUV by following the stability of the asymmetry over time with fluorescence spectroscopy measurements. Figure 4c shows confocal images taken 22 h after vesicle formation, before and after addition of sodium dithionite quencher. In contrast with vesicles visualized after 6 h, some residual fluorescence on the membrane could be observed, suggesting lipid flip‐flop to the polymer leaflet. One should note that the diffusion rates are attributed to the PE‐NBD lipid across the polymer–lipid bilayer. We could estimate by fluorescence spectroscopy that 75% of the initial fluorescence signal on the outer monolayer was lost following the addition of quencher after 22 h (Figure 4d). We then followed the kinetics of lipid flip‐flop to estimate the half‐life for POPC to move from the outer to the inner side of the membrane, assuming that the dye exhibits the same fluorescence on both sides of the membrane. Therefore, the loss of fluorescence was assumed to be directly related to the amount of POPC diffusing in the inner leaflet. Figure 4e shows the evolution of remaining POPC on the outside of the membrane (POPC (out)) after several hours and allowed us to determine a half‐life around 7.5 h, a value that is consistent with previously reported values of 5–6 h for giant asymmetric lipid vesicles.^44^

**Figure 5:**
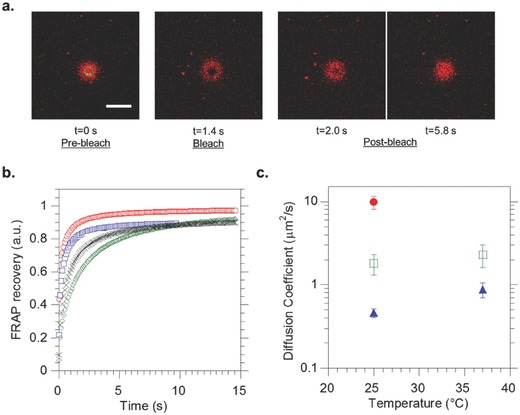
FRAP measurements on DOPE‐rhodamine inserted into the membrane of PBut_2.5_‐*b*‐PEO_1.3_ and asymmetric POPC/PBut_2.5_‐*b*‐PEO_1.3_ (aGHUV) giant vesicles. a) Confocal images of a vesicle during the three phases of FRAP experiment, namely prebleach, bleach, and postbleach. We observe the recovery of fluorescence inside the ROI with time. Scale bar: 10 µm. b) Normalized fluorescence intensity profiles during recovery. Pure PBut_2.5_‐*b*‐PEO_1.3_ giant vesicles (25 °C green, 37 °C black), POPC/PBut_2.5_‐*b*‐PEO_1.3_ aGHUV (25 °C red, 37 °C blue). c) Measured lipid lateral diffusion coefficients (*D* (µm^2^ s^−1^)) for different membranes and temperatures: pure POPC giant vesicles (●) (ref. 39), POPC/PBut_2.5_‐*b*‐PEO_1.3_ aGHUV (◻), pure PBut_2.5_‐*b*‐PEO_1.3_ giant vesicles (▲). Measurements were performed and averaged on 5–10 vesicles for each system.

In addition, it is well established that lipid transverse diffusion across the cell's bilayer membrane occurs at much slower rates than translational diffusion, i.e., the motion of lipids in one monolayer, which was first evidenced in 1970.^68^ Translational or lateral diffusion has been quantified in synthetic membrane systems using the standard fluorescence recovery after photobleaching (FRAP) technique which consists in measuring the recovery of fluorescence in a determined region of interest of a membrane (ROI) that was exposed to photobleaching.^69^ The motion of unbleached lipids in the membrane allows a recovery of fluorescence in the bleached ROI with kinetics that can be monitored and fitted with appropriate models. Lateral diffusion coefficients in the literature for pure PBut‐*b*‐PEO or pure POPC giant vesicles, were reported to be respectively *D* = 0.22 and 9.8 µm^2^ s^−1^.^61^ Using giant hybrid unilamellar vesicles presenting homogenous distribution of the lipid and polymer content at the microscale, it was shown that lipid lateral diffusion coefficient could be modulated by the lipid/polymer composition. We thus hypothesized that an asymmetric POPC/PBut‐*b*‐PEO membrane would give intermediate diffusion values closer to those observed in biological membranes which are around 1 µm^2^ s^−1^.^70^ We used confocal microscopy imaging (**Figure** **5**a) and the FRAP analyzer software to measure the diffusion coefficients of a DOPE‐rhod probe inserted into the POPC outer monolayer of the aGHUV for different temperatures and different membrane types. Rhodamine was chosen as a fluorescent probe because of its photostability as compared with other dyes. After collecting the intensity profiles from the confocal images, we analyzed the results with the FRAP software. The data were first double‐normalized to remove the fluorescence variations between samples, by taking into account the background fluorescence and the slow bleaching of the dye during fluorescence recovery at low laser intensity. We used the circular spot diffusion model described by Equation (1) to fit the data

(1) $$\mathrm{FRAP}\left(t\right)\;\;=\;\;a_{0}+a_{1}.e^{-\frac{\tau }{2\left(t-t_{\mathrm{bleach}}\right)}}.\left(I_{0}\left(\frac{\tau }{2\left(t\;-\;t_{\mathrm{bleach}}\right)}\right)\;\;+\;\;I_{1}\left(\frac{\tau }{2\left(t\;-\;t_{\mathrm{bleach}}\right)}\right)\right)$$

with $\tau =\frac{\omega^{2}}{D}\;$, where ω is the radius of the bleach spot, *D* the diffusion coefficient, *a*_0_ and *a*_1_ normalizing coefficients, and *t*_bleach_ the bleach time.

As can be seen in Figure 5a, FRAP measurements were performed at the top of the giant vesicles that consequently appear as fluorescent disks under confocal observation. The experiment can be decomposed into three different phases: the prebleach phase where the ROI chosen on the top of the vesicles is irradiated at low laser power, followed by a defined bleaching phase at high laser power and a longer postbleach phase to monitor the recovery of fluorescence inside the ROI induced by the diffusion of lipids in the POPC outer leaflet. Figure 5b shows the obtained intensity profiles during the postbleach phase after normalization. These data were fitted with the model described previously to obtain the lateral diffusion coefficients (*D*, µm^2^ s^−1^) of the probe in the lipid monolayer (Figure 5c). For POPC/PBut_2.5_‐*b*‐PEO_1.3_ aGHUV we found *D* = 1.8 ± 0.50 µm^2^ s^−1^ for DOPE‐rhod at 25 °C. Under the same experimental conditions, the diffusion coefficient for DOPE‐rhod in pure PBut_2.5_‐*b*‐PEO_1.3_ giant vesicles was found to be *D* = 0.46 ± 0.055 µm^2^ s^−1^ and it has been shown that for pure POPC giant vesicles *D* = 9.8 ± 1.7 µm^2^ s^−1^.^61^ Therefore, as for GHUV, an intermediate diffusion coefficient value is found, and shows that despite the asymmetric character of the membrane, the lipid lateral diffusion coefficient is lowered by the copolymer chains, suggesting some interdigitation between lipids and copolymer chains in the membrane. The lipid lateral diffusion has been also evaluated in aGHUV at 37 °C and is found to be 2.3 ± 0.7 µm^2^ s^−1^ confirming the impact of temperature on membrane's fluidity,^30^, ^71^, ^72^ and the relevance of our systems, with a lateral diffusion coefficient close to those reported in cells' membrane.

## 3 Conclusions

Overall, our technique provides a method for controlled assembly of giant vesicles exhibiting an asymmetric lipid‐polymer membrane that can easily be tuned. The confocal observations and the quenching experiments strongly suggest the presence of an asymmetric membrane, whose composition and structure slowly evolve during time as illustrated by “flip flop” experiments. Lateral diffusion coefficients of the lipid in the vesicle were found to be intermediate between those of pure lipid or pure polymer membranes and support the presence of an asymmetric membrane.

In addition to aGHUV with an outer leaflet of tunable lipid type and inner leaflet of copolymer, we also showed that the reverse asymmetric structures with the lipid leaflet facing the inside of the vesicle could be obtained. The total asymmetry was proven using a fluorescence‐quenching assay. Interestingly, the lateral lipid diffusion coefficient is perturbed by the presence of the copolymer chains, probably because of slight interdigitation between the two leaflets, leading to a lateral diffusion coefficient comparable to the ones known for lipids in biological cells. The originality of our approach was to prepare a mix‐system combining lipid and polymer advantages to afford a cell‐sized giant vesicle with an asymmetric membrane. As compared with previously reported lipid/lipid or polymer/polymer asymmetric membranes, asymmetric hybrid polymer/lipid membrane represents an alternative toward preparing model systems for cell biomimicry.

## 4 Experimental Section

*Materials*: The phospholipids used for the liposomal systems were POPC and DMPC. The dyes used were PE‐NBD (ammonium salt) and DOPE‐rhod (ammonium salt). These materials were purchased from Avanti Polar Lipids Inc., (Albaster, AL, USA) and used without further purification. PBut2.5‐*b*‐PEO1.3 was ordered from Polymer Source (P18422‐BdEO, *M*_w_/*M*_n_ 1.04, 89% 1,2‐addition of butadiene). All other solvents and reagents used were of analytical grade and purchased from Sigma‐Aldrich Chemical Co.

*Methods*: Fluorescence experiments were carried out on a Spectra Max M2 microplate spectrophotometer (Molecular Devices). Laser scanning confocal microscopy images were acquired on an inverted Leica TCS SP5 microscope equipped with an HCX PL APO 63×, NA 1.4 oil immersion objective in fluorescence mode. Samples (≈20 µL) were injected in a homemade chamber that was sealed to prevent evaporation. The laser outputs were controlled via the Acousto‐Optical tunable filter and the two collection windows using the Acousto‐Optical beam splitter and photomultipliers as follows: NBD was excited with an argon laser at 488 nm and DOPE‐rhod was excited at 561 nm. The helium–neon laser at 633 nm (10 %) was used in transmission mode.

*Fluorescence Recovery after Photobleaching*: FRAP was performed using the FRAP‐Wizard of the LAS‐AF microscope software which allowed to control and tune the scanning conditions: prebleach, photobleach, and postbleach phases. DOPE‐rhod was excited and bleached with the 561 nm laser line and the emission was collected in the 600–700 nm range. ROIs were defined over the vesicles with a diameter of 3 µm. FRAP acquisition was started with ten images scan at low (3–5%) laser power. Then, the dye was bleached locally inside the ROIs at 100% laser power using a scan of three frames. Finally, fluorescence recovery was monitored by the acquisition of a series of 150–200 images at the same low laser power as the prebleach phase. The images were acquired with a 6× zoom, using a 256 × 256 pixel frame and bidirectional scan at a 1400 Hz line frequency speed. The pinhole was set to 222.92 µm (2 Airy). To control the temperature, the microscope was equipped with a heating and cooling stage (PE120XY stage size 160 × 116 mm) from Linkam Scientific Instruments, UK, with temperature range: −25 to 120 °C, heating/cooling rate: 0.1 to 20 °C min^−1^, and control and stability: +/−0.1 °C. FRAP experiments were performed at 25 or 37 °C. The FRAP analyzer software was used for quantitative analysis of the FRAP data. After normalization (double normalization), the data were fitted with the circular spot model in 2D diffusion. It is important to note that the preparation method used to generate aGHUV, described below, allows the sedimentation of the vesicles in the bottom of the cover slip and perfect immobilization, obviously necessary for FRAP experiments.

*Preparation of Asymmetric Giant Hybrid Unilamellar Vesicles*: Asymmetric giant hybrid unilamellar PBut_2.5_‐*b*‐PEO_1.3_—lipid vesicles (aGHUV) were prepared by a previously reported emulsion‐centrifugation method.^58^ Briefly, 5 µL of a sucrose solution (0.3 m sucrose, 0.01 m HEPES, 0.15 m NaCl, pH 7.4) was poured into 3 mg mL^−1^ PBut_2.5_‐*b*‐PEO_1.3_ in 500 µL toluene. The solution was vigorously hand‐shaken for 30 s to create a water‐in‐oil emulsion. An interface was prepared by pouring 30 µL of the desired lipid (1.5 mg mL^−1^) in toluene over 30 µL glucose solution (0.3 m glucose, 0.01 m HEPES, 0.15 m NaCl, pH 7.4) and allowed to stabilize for 2 h. 75 µL of the above emulsion was slowly poured over the interface and the sample was immediately centrifuged (3 min, 500 g) at the same temperature the interface was formed. The resulting aGHUV were recovered in the lower phase. For fluorescence quenching experiments, 0.15 mol% PE‐NBD (or DOPE‐rhod) was added to the lipid solution. The fluorescence intensity was monitored over time on a spectrophotometer (λ_exc_ = 488 nm) and data were normalized with the average fluorescence obtained for unquenched vesicles. For the preparation of reverse asymmetric vesicles, an interface was formed and left to rest for 30 min with 3 mg mL^−1^ PBut‐*b*‐PEO. The emulsion was prepared with different concentrations of lipid and sonicated 10–15 s in a bath sonicator.

## Conflict of Interest

The authors declare no conflict of interest.

## Supporting information

Supplementary

Supplementary

Supplementary
