# Supplementary material for: Asymmetric Hybrid Polymer–Lipid Giant Vesicles as Cell Membrane Mimics
Source: Adv Sci (Weinh). 2017 Dec 5;5(1):1700453. doi: 10.1002/advs.201700453 (PMC5770682; doi:10.1002/advs.201700453)
Supplement: Supplementary file 1 — Supplementary [file ADVS-5-na-s002.pdf]

Copyright WILEY-VCH Verlag GmbH & Co. KGaA, 69469 Weinheim, Germany, 2017.

ADVANCED SCIENCE
Open Access

Supporting Information

for *Adv. Sci.,* DOI: 10.1002/advs.201700453

Asymmetric Hybrid Polymer–Lipid Giant Vesicles as Cell Membrane Mimics

*Ariane Peyret, Emmanuel Ibarboure, Jean-François Le Meins, and Sebastien Lecommandoux**

Copyright WILEY-VCH Verlag GmbH & Co. KGaA, 69469 Weinheim, Germany, 2016.

Supporting Information

Asymmetric Hybrid Polymer-Lipid Giant Vesicles as Cell Membrane Mimics

Ariane Peyret, Emmanuel Ibarboure, Jean-François Le Meins, Sebastien Lecommandoux *

Laboratoire de Chimie des Polymères Organiques, LCPO, Université de Bordeaux, CNRS, Bordeaux INP, UMR 5629, 16 Avenue Pey Berland F-33600 Pessac, France
E-mail: lecommandoux@enscbp.fr

Video S1: 3D-reconstruction from confocal observations of POPC/$PBut_{2.5}$-*b*-$PEO_{1.3}$ asymmetric vesicles. The membrane is tagged with DOPE-rhodamine. Videos are showing a perfect homogenous repartition of the lipids on all the vesicle surface (diameter about 20 microns).

Video S2: Z-stack representation from confocal observations of POPC/$PBut_{2.5}$-*b*-$PEO_{1.3}$ asymmetric vesicles. The membrane is tagged with DOPE-rhodamine. Videos are showing a perfect homogenous repartition of the lipids on all the vesicle surface (diameter about 20 microns).

Preparation of $PBut_{2.5}$-b-$PEO_{1.3}$ (outer layer) / POPC (inner layer) reverse asymmetric vesicles.

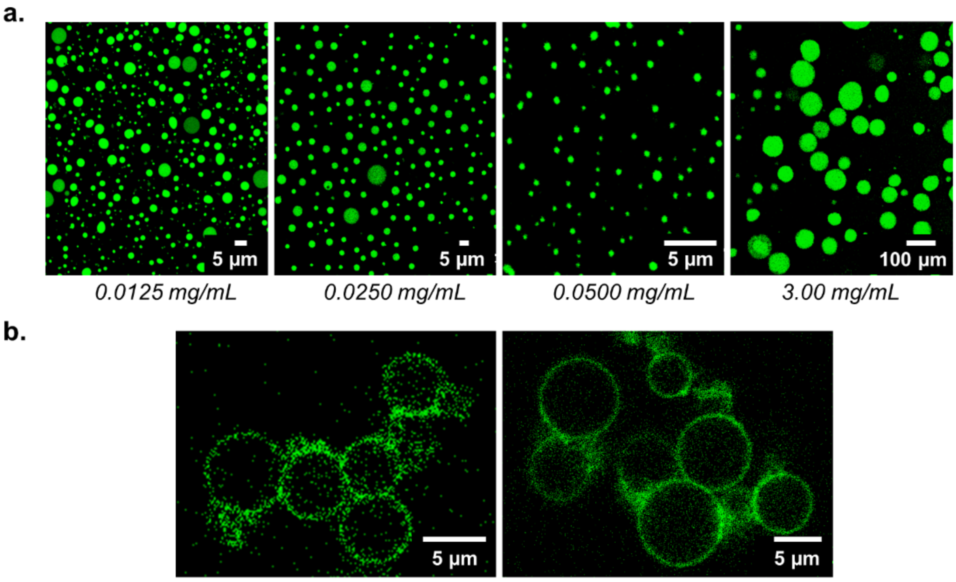

Figure S1. Asymmetric $PBut_{2.5}$-*b*-$PEO_{1.3}$ (outer leaflet) / POPC (inner leaflet) vesicles. Confocal images of a) POPC stabilized emulsions of sucrose droplets (0.1 mg/mL fluorescein) in toluene for different POPC concentrations and b) asymmetric PBut-*b*-PEO / POPC vesicles.

To prepare the reverse asymmetric vesicles (see Figure 1, route b)) we first had to optimize the emulsion conditions, since obtaining a stable homogeneous emulsion with a lipid turned out to be more complex than with PBut-*b*-PEO. Different concentrations of POPC were tested and fluorescein was added to the sucrose solution to visualize the droplets under confocal microscopy, as shown in Figure S1. a). Using 0.025 mg/mL POPC gave the most stable emulsion with droplet sizes ranging from 4 to 6 µm approximately after sonication. Other concentrations either gave inhomogeneous droplets (0.0125 mg/mL) or too small or too large sizes (respectively 0.050 mg/mL and 3.0 mg/mL). Figure S1. b) shows the resulting reverse asymmetric polymer / lipid vesicles. The sizes (about 5 µm) are consistent with the sizes measured for the emulsion droplets which led us to assume that we did form the reverse vesicles.

DMPC (outer layer) / PBut$_{2.5}$-b-PEO$_{1.3}$ (inner layer) asymmetric vesicles

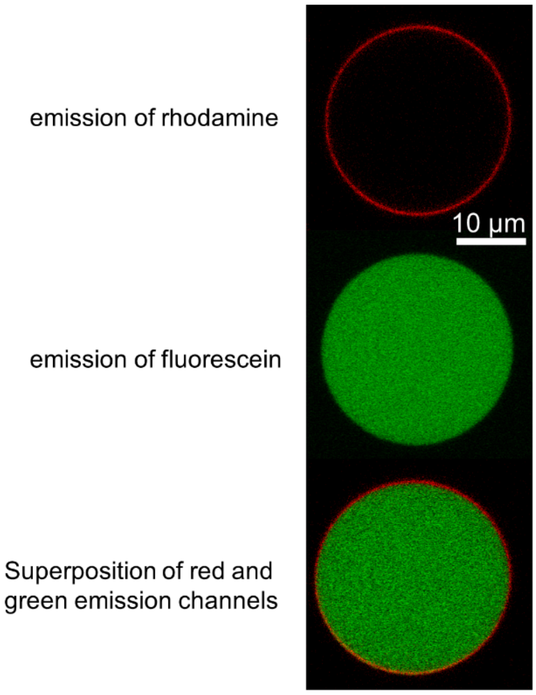

Figure S2. Confocal observation of DMPC/PBut$_{2.5}$-*b*-PEO$_{1.3}$ asymmetric vesicles. The membrane is tagged with DOPE-rhodamine (red) and the vesicles are loaded with fluorescein (green). Top: emission of rhodamine; middle: emission of fluorescein; bottom: overlay.
